# A novel technique with Dacron vascular graft augmentation for knee extensor mechanism repairs: Technical note

**DOI:** 10.1051/sicotj/2022034

**Published:** 2022-08-15

**Authors:** Rómulo Silva, Eva Campos Pereira, Marco Distefano, Roskams Toon, Jeroen Verhaegen, Koen Lagae, Peter Verdonk

**Affiliations:** 1 Unidade Local de Saúde do Alto Minho 4904-858 Viana do Castelo Portugal; 2 Hospital Central do Funchal 9000-177 Madeira Portugal; 3 Università degli Studi di Firenze, AOU Careggi 50121 Firenze Italy; 4 Faculty of Medicine and Life Sciences, University of Antwerp 2610 WILRIJK Antwerp Belgium; 5 Orthoca, AZ Monica 2100 Antwerp Belgium

**Keywords:** Knee, Extensor mechanism, Reconstruction, Graft

## Abstract

Ruptures of the extensor apparatus can have different etiologies and be complicated by underlying situations. Direct repair is not always possible, and reconstruction procedures can be insufficient, which leads to the appearance of multiple augmentation techniques to improve the strength of these constructs. Despite the proven results of these techniques, numerous procedures are described without any gold standard. We present our augmentation method for repairing the knee extensor apparatus with a vascular prosthesis that facilitates healing, does not interfere with the primary procedure, has no donor morbidity or rejection risk, and allows earlier mobilization and rehabilitation. The technique was used in different cases with multiple etiologies that needed reinforcement, with promising results.


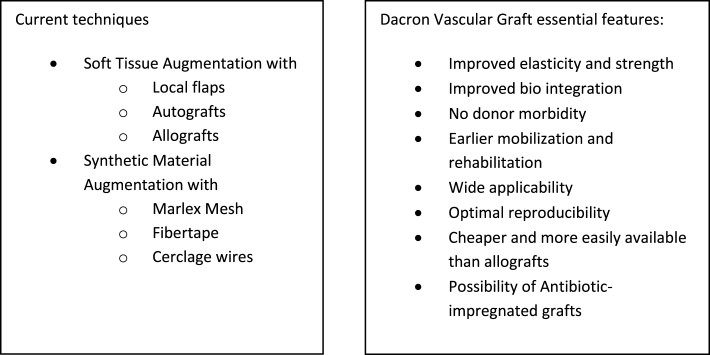


## Introduction

Although the rupture of the extensor mechanism of the knee is an uncommon presentation in orthopedic practice, it is associated with elevated morbidity and disability. The mechanism of injury can be traumatic or atraumatic. Direct trauma is most common, while atraumatic ruptures occur mainly in immunocompromised patients with chronic metabolic diseases, prolonged use of corticosteroids, periprosthetic infections, or iatrogenic causes [1].

These injuries are particularly challenging to address in a total knee replacement (TKR) and can occur in any element of this complex structure: quadriceps muscle and tendon, patella, or patellar tendon [2].

Surgical management is mandatory since the conservative treatment, long leg bracing in extension, is associated with poor results and severe disabilities [1, 2].

Several techniques have been described ranging from direct repair (with or without augmentation) to reconstruction (with biological or synthetic grafts) [2, 3].

Isolated primary repairs, with several described techniques [4], show high rates of failure and are not always feasible, especially in chronic or massive ruptures [5, 6]. In these cases, different problems arise, such as tendon retraction, muscle atrophy, poor soft tissue quality, extensive fibrosis or adhesions, and poor bone stock [4].

Such cases can require complex reconstructive techniques which demand an allograft, autograft, synthetic material, local flap, or a combination of procedures [2]. Augmentation with artificial tissues increases the strength of the constructs, decreases gap formation across the repair site, and allows for fibrous tissue ingrowth [6, 7]. Advantages of this type of material include simplicity of use, absence of donor site morbidity and disease transmission, no restrictions regarding regulations, options with antibiotic-impregnated grafts in infection-related cases, and lower costs when compared to allografts [8].

## Technique

**Figure 1:**
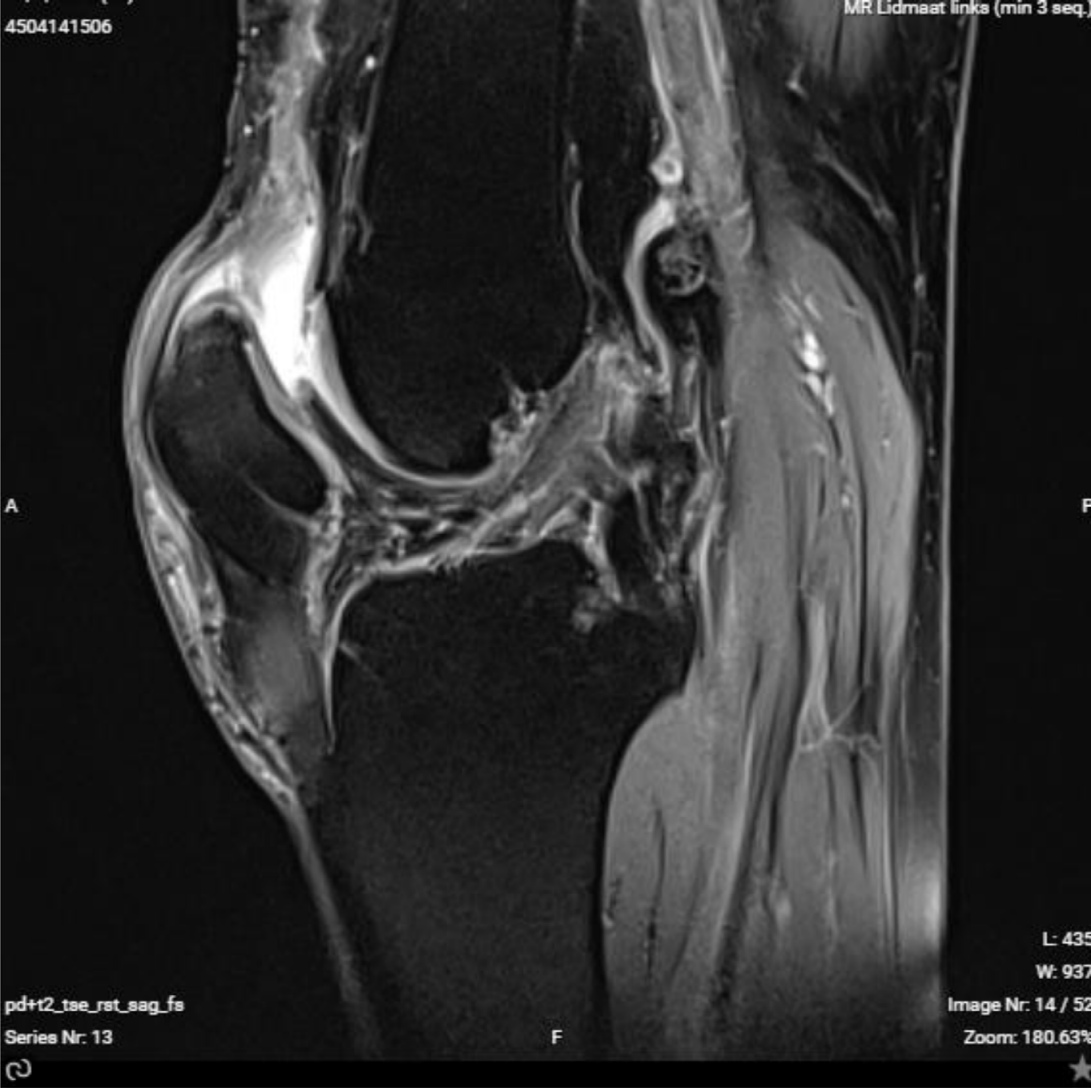
MRI showing the quadriceps lesion.

**Figure 2:**
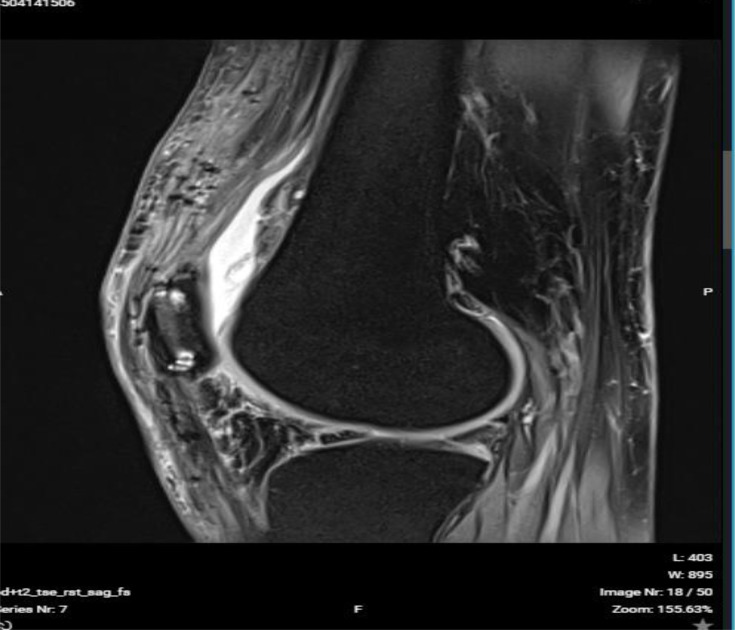
Post operative MRI: Notice the improvement of the patellar height and quadriceps fibrosis.

**Figure 3:**
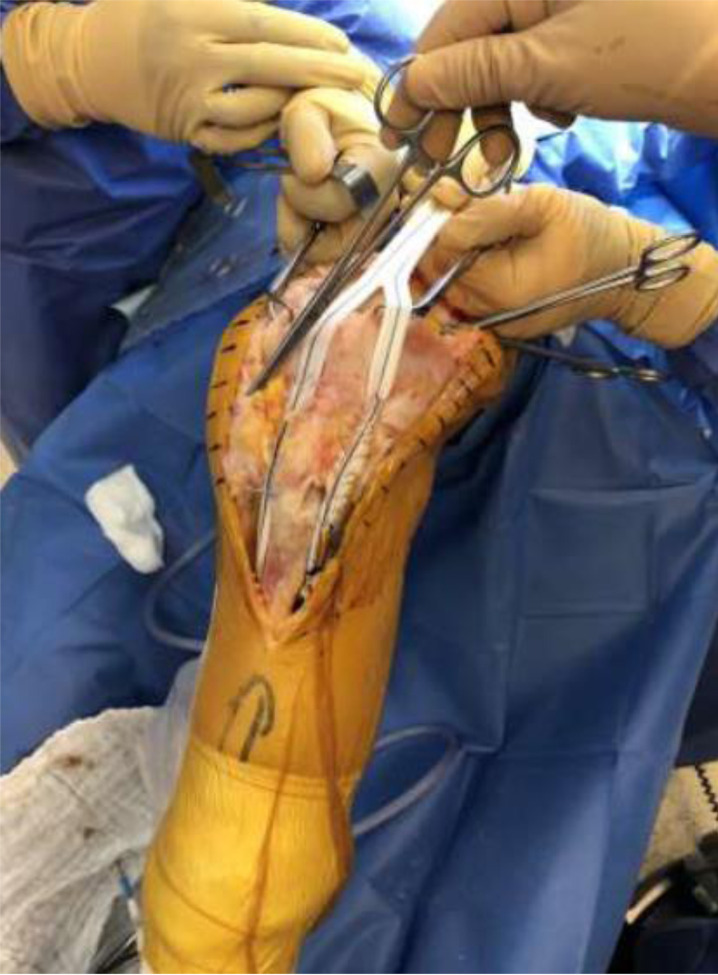
Augmentation of the allograft with the vascular graft.

**Figure 4:**
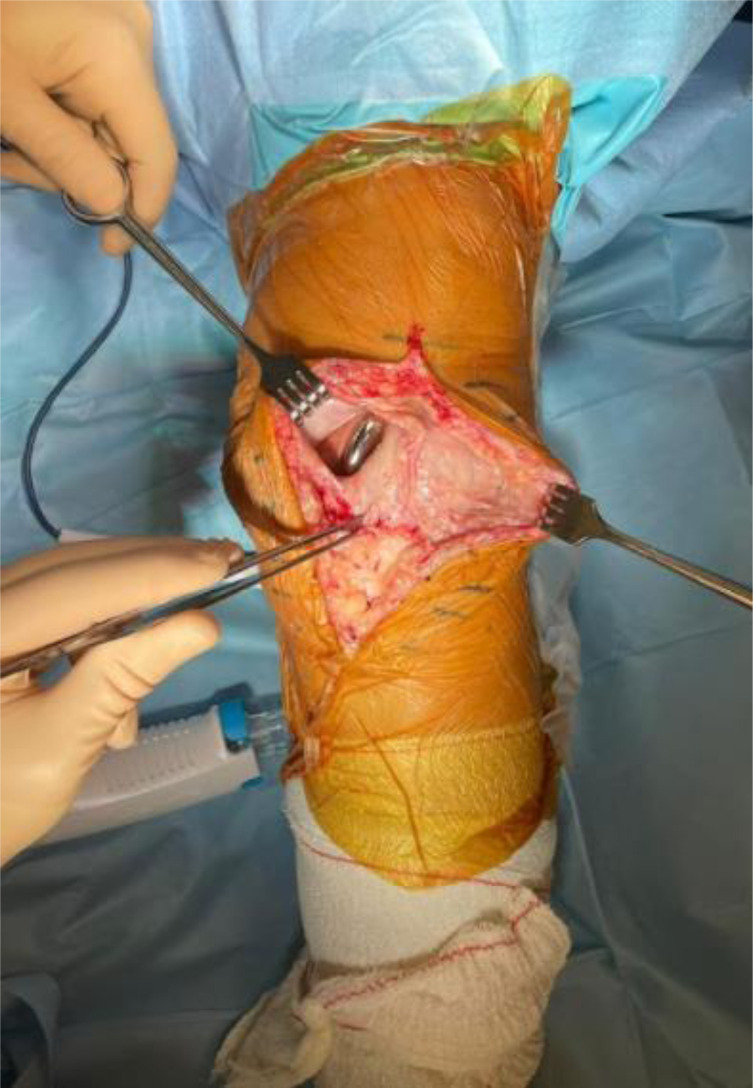
Medial retinaculum defect and quadriceps chronic degeneration.

**Figure 5:**
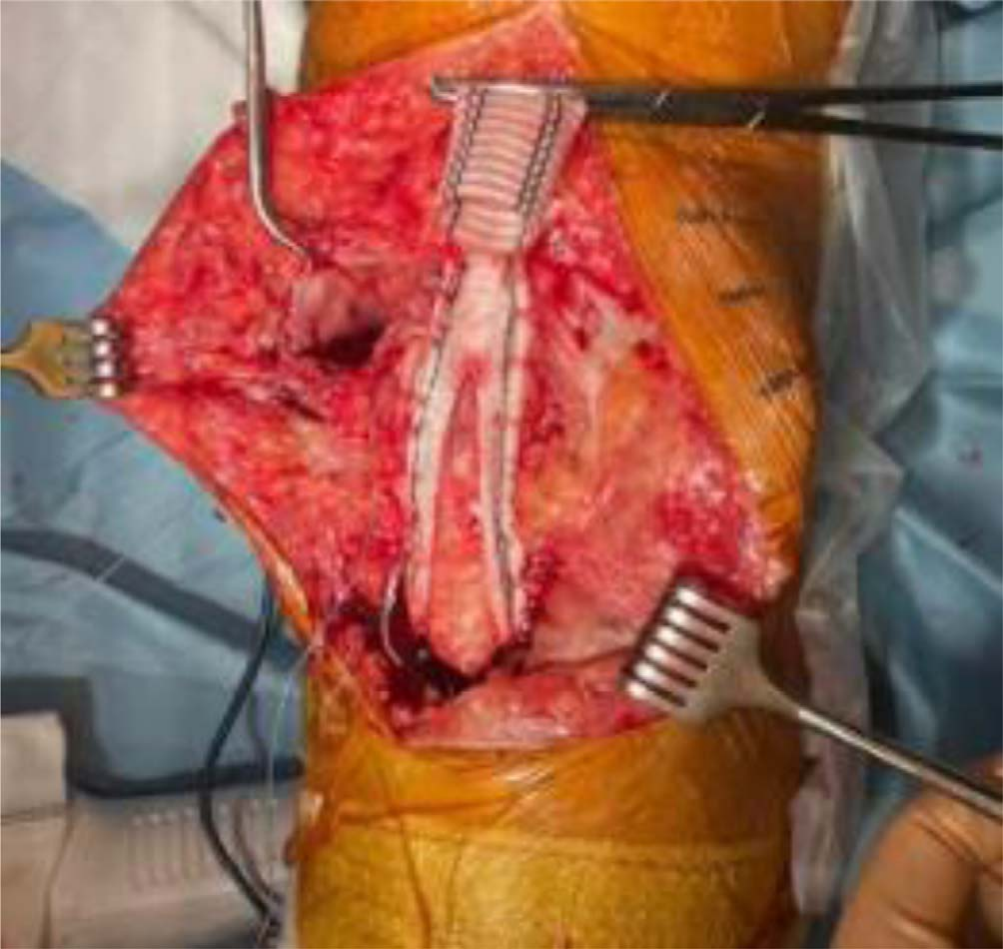
Augmentation with the Dacron vascular graft.

**Figure 6:**
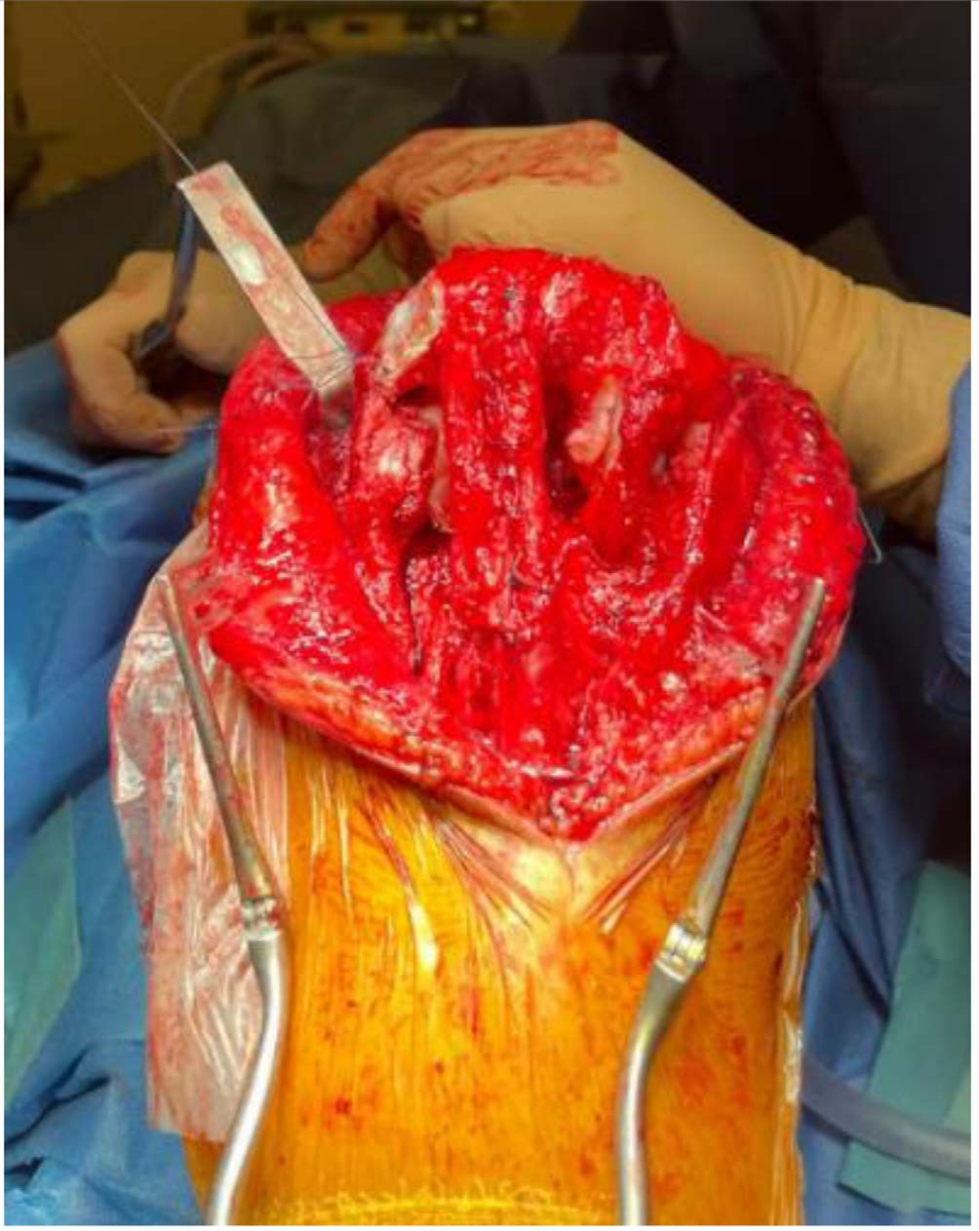
Patellar tendon suture and reinforcement.

We present our technique for augmentation on repairs of the extensor apparatus with AlboGraft^®^ Polyester Vascular Graft (https://www.lemaitre.com/products/albograft-polyester-vascular-grafts), but any similar bifurcated vascular graft is applicable. We have applied it to different indications and circumstances (Table 1), such as: chronic quadriceps rupture associated with patella baja and quadriceps retraction with a failed primary repair – performed a Z plasty of the patellar tendon, which allowed direct suturing of the quadriceps tendon, followed by augmentation with the vascular graft for earlier rehabilitation (Figures 1 and 2); bilateral extensor mechanism failure after TKR treated with extensor apparatus allograft, which failed on one side – performed reconstruction of the extensor apparatus with the vascular graft and an augmentation on the contralateral side to protect and reinforce the allograft (Figure 3); chronic extensor mechanism failure with medial extension and recurrent patellar dislocation after TKR with failed direct repair (Figure 4) – performed a revision of the direct repair with an associated augmentation with the vascular graft and remplissage of the medial retinaculum (Figure 5); patellar tendon rupture – performed a direct suture and augmentation (Figure 6), which is the case used in the technique pictures. This wide range of applications in extensor apparatus pathology is one of the main advantages of our procedure.

**Table 1:** Some of the complicated cases this technique was developed to solve.

Patient	Age	Status	Surgery	Post-Operative	Follow up time	Patient specifics	Outcomes
Female	49	TKR Quadriceps tendon suture Insufficiency of the extensor apparatus with patella baja and patellar instability Complete avulsion of the vastus medialis and medial retinaculum	Suture of the quadriceps, vastus medialis and retinaculum with augmentation with the vascular graft. Tibial tuberosity transfer for patellar tracking improvement	6-week full extension brace	2 years	Heavy smoker	Full Extension Patellar stability 90° Flexion
Male	75	Chronic rupture of quadriceps tendon Patella baja with patellar tendon shortening Failed primary repair surgery	Z plasty of the patellar tendon + reinsertion of the quadriceps tendon in the patella with augmentation with the vascular graft.	6-week full extension brace	2 years		Full extension No pain Autonomous Flexion deficit (75°)
Female	60	TKR TKR revision Failed allograft reconstruction of extensor apparatus for bilateral quadriceps rupture Laxity of the contralateral extensor allograft reconstruction	Reconstruction of the extensor apparatus with vascular graft + retensioning and augmentation of the allograft with the vascular prosthesis	3 months extension brace	3 years	Multiple surgeries on the knee	Full extension Straight leg raising Flexion deficit (70°) Re-establishment of extensor strength on the other side
Female	63	Patellectomy following patellar fracture Extensor lag Decreased ROM	Extensor apparatus augmentation with vascular graft	Ottobock brace for progressive ROM for 6 weeks	13 months		Improved extensor lag and sense of stability Straight leg raising ROM −5 to 115°
Female	52	Patellar tendon rupture	Direct suture with augmentation with vascular graft	6-week full extension brace	3 months		Full ROM Full recovery

**Figure 7:**
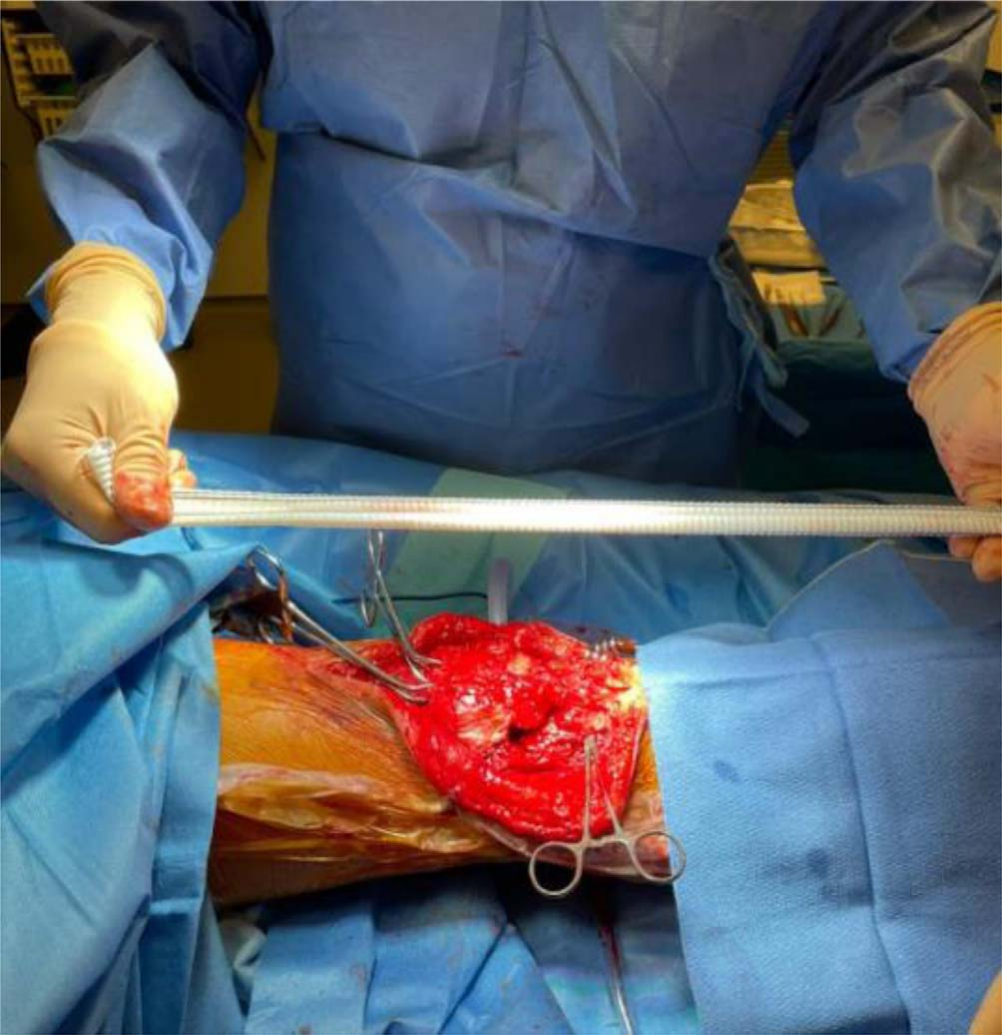
Tensioning of the vascular graft.

The patient is prepared in the usual sterile fashion while the vascular graft (20.2 × 9.1 mm polyester vascular bifurcated prosthesis with 50 cm of total length – *AlboGraft*^*®*^ is tensioned (Figure 7).

A midline knee incision is used to approach the injury (previous incision is used if present), followed by a careful soft tissue debridement and identification of the quadriceps tendon, vastus muscle bellies, patellar tendon, and anterior tibial tuberosity.

**Figure 8:**
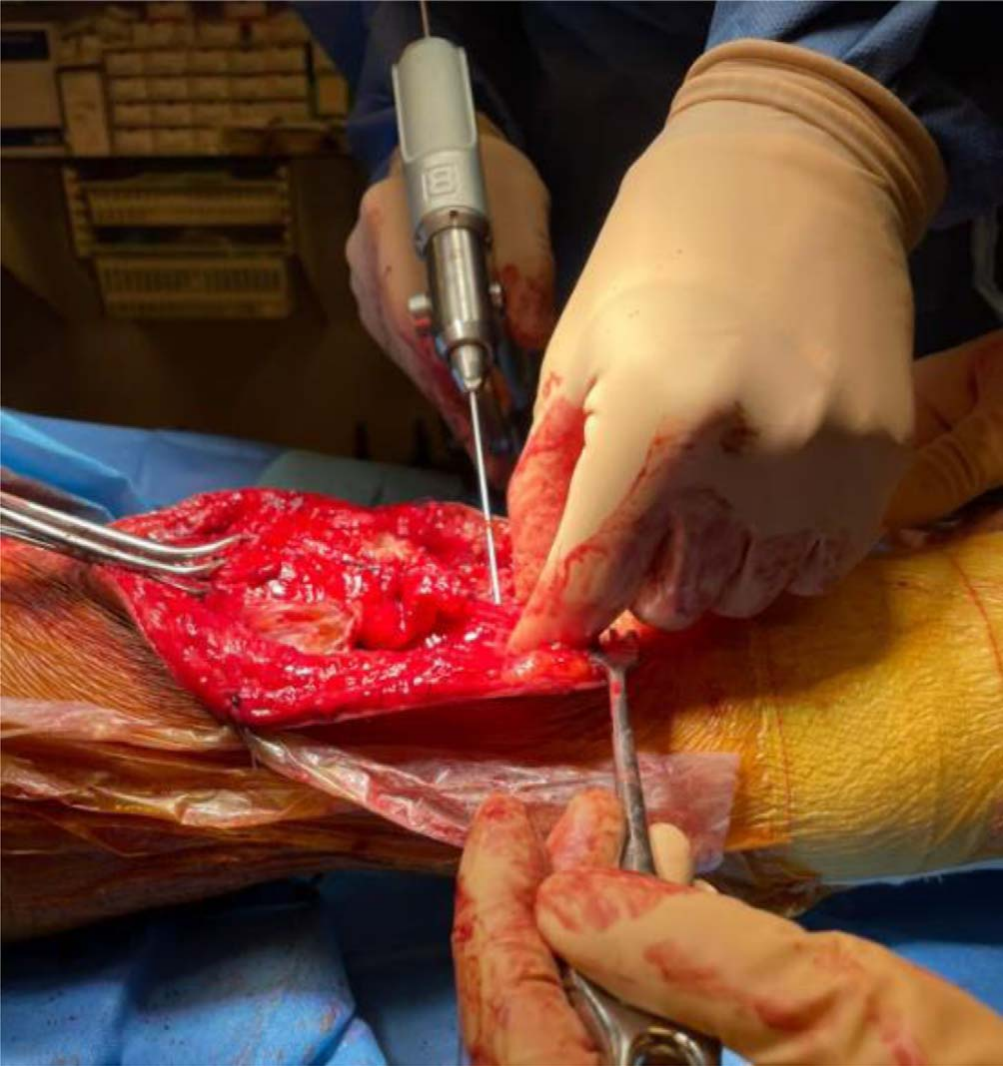
2.8 mm guidewire is inserted transversely through the distal 1/3 of the anterior tibial tuberosity.

**Figure 9:**
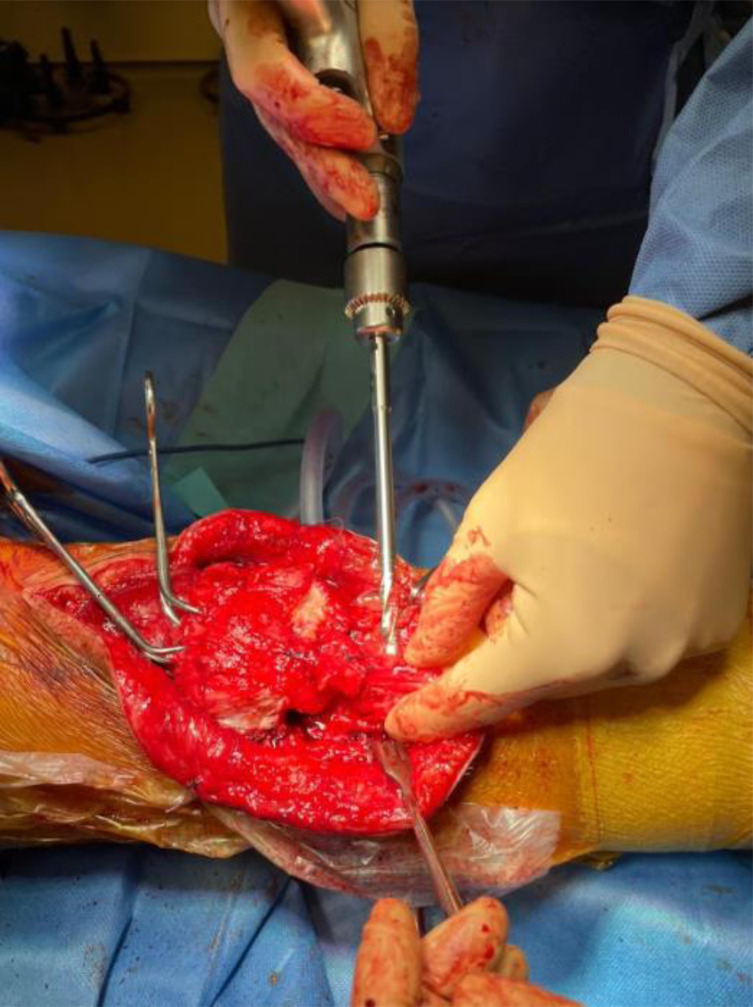
7 mm cannulated drill is passed through the 2.7 mm guidewire.

**Figure 10:**
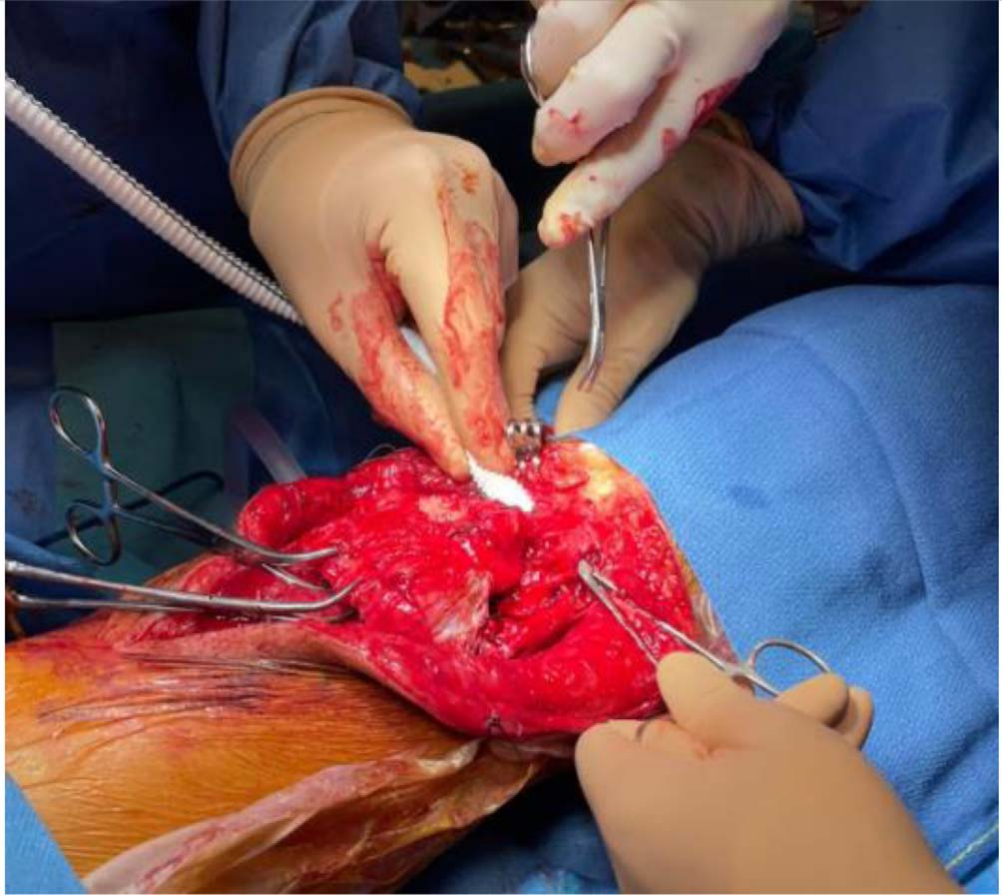
A Kocher clamp is used to pass one leg of the bifurcation through the 7 mm tunnel.

**Figure 11:**
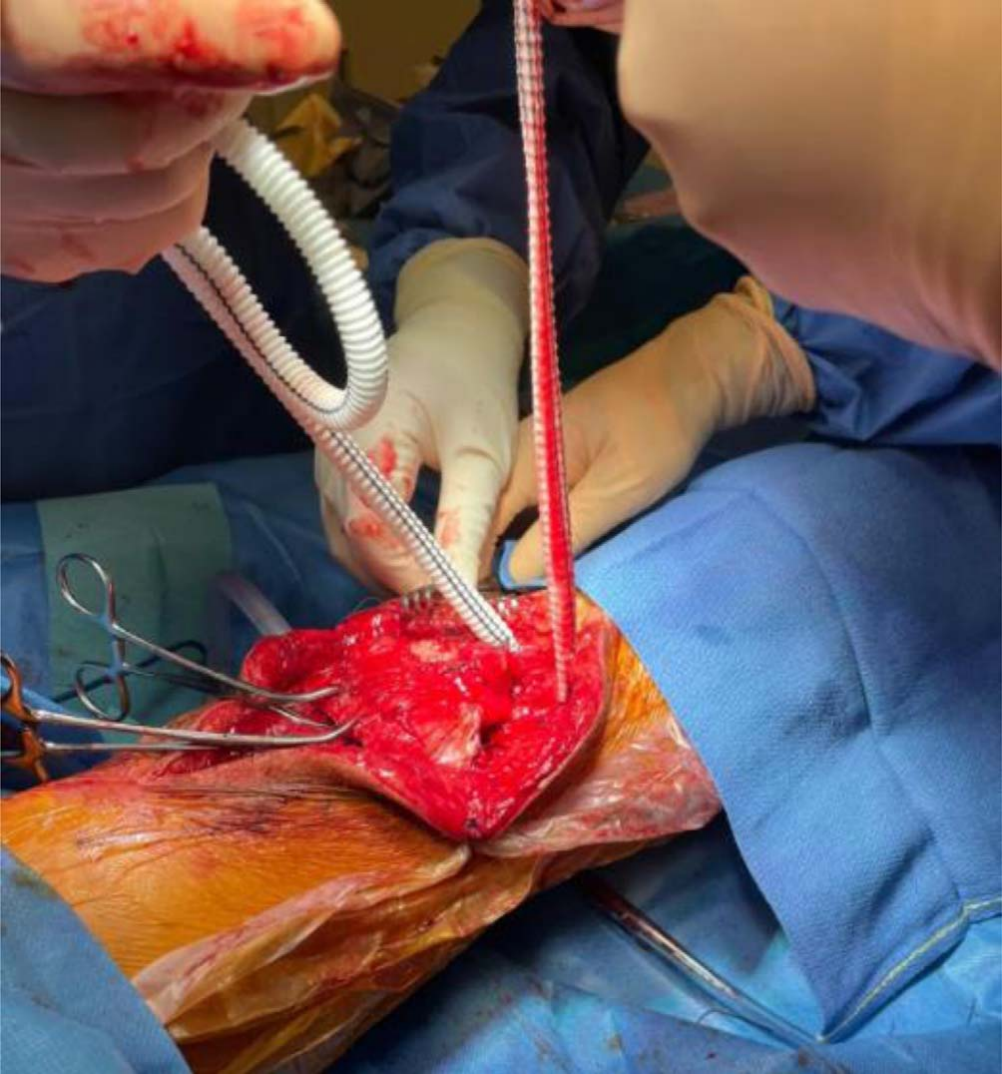
One leg of the vascular graft’s bifurcation is passed through.

**Figure 12:**
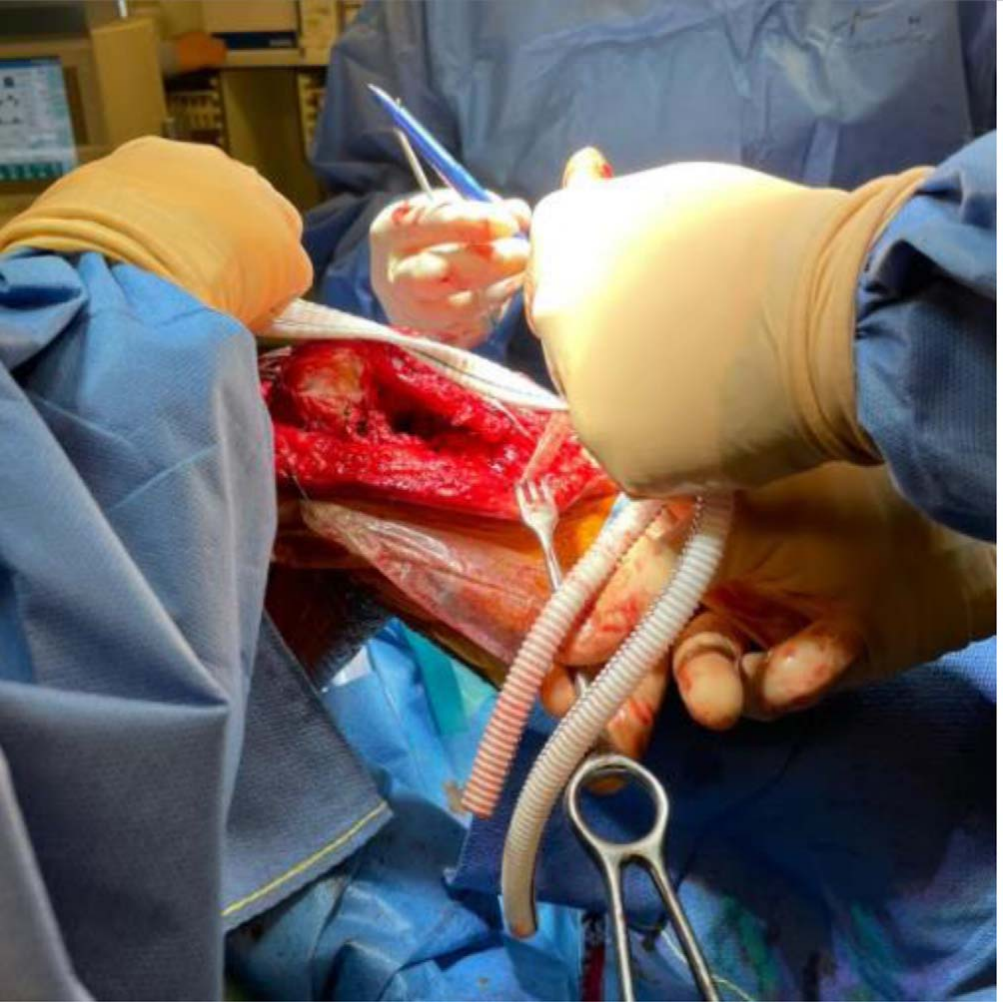
Performing the square knot between both ends of the bifurcation.

**Figure 13:**
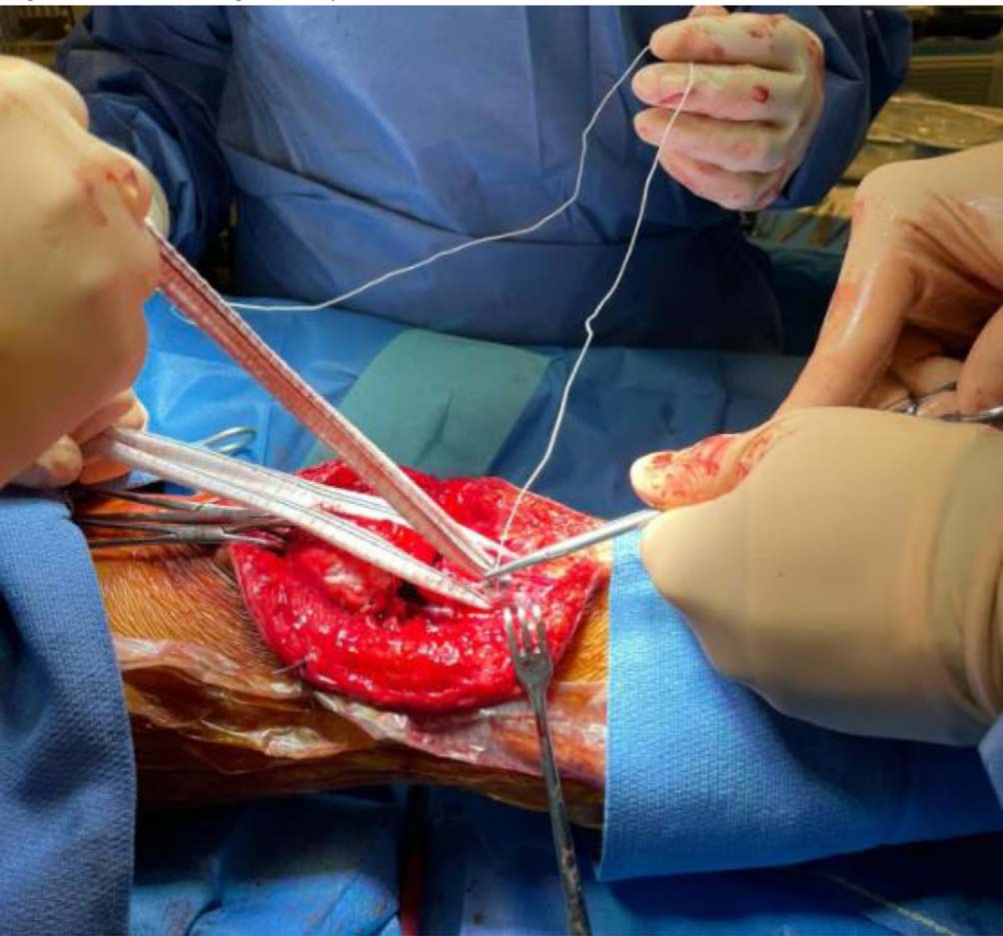
Reinforcement of the knot with non-absorbable sutures.

**Figure 14:**
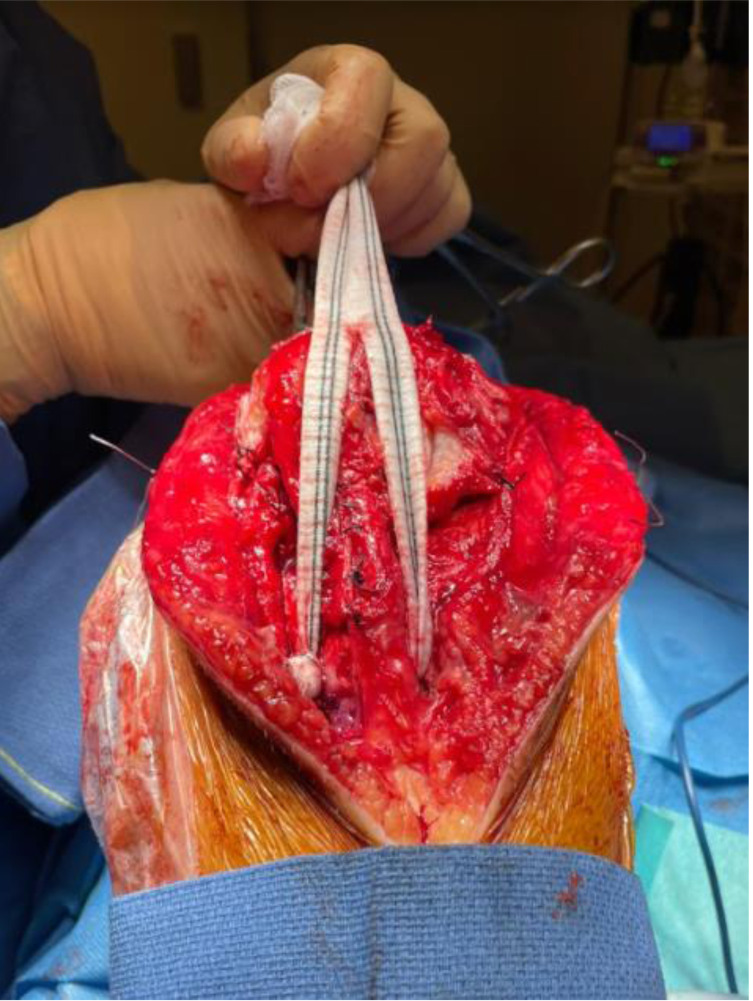
The vascular graft is oriented along the extensor apparatus, embracing the patellar tendon from both sides, while converging at a midpatellar level.

**Figure 15:**
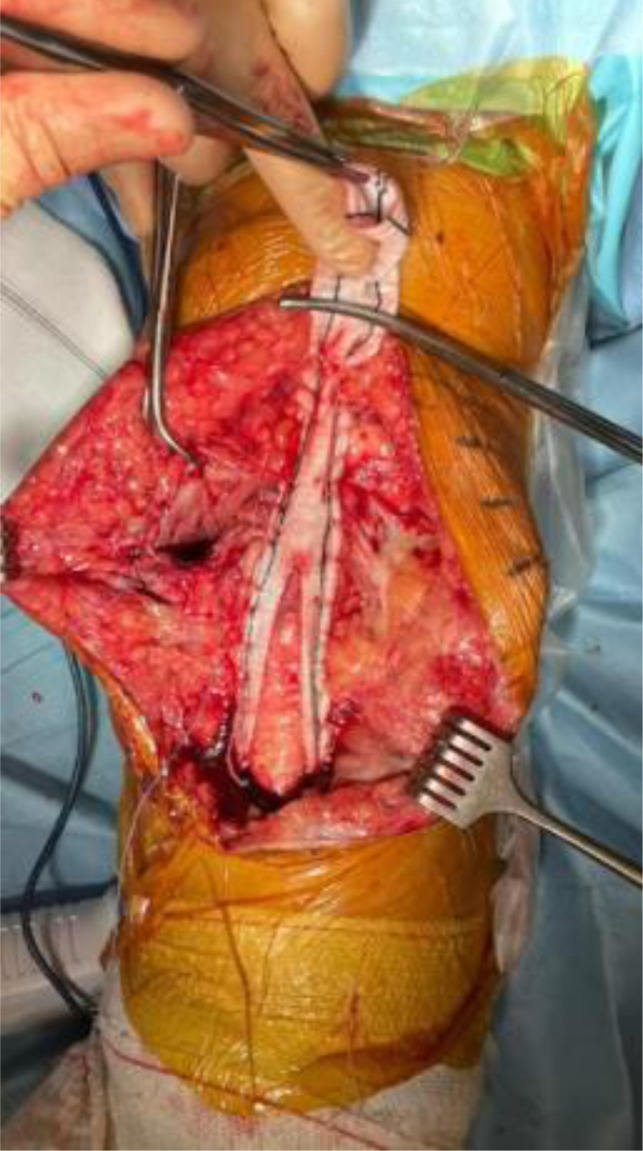
Suturing the graft along the extensor apparatus.

A 2.8 mm guidewire is carefully placed at the lower third of the tibial tuberosity (Figure 8), followed by a tunnel with a 7 mm cannulated drill (Figure 9), one of the legs of the bifurcated graft is passed through (Figures 10 and 11), then both ends of the bifurcation are tied together with a square knot (Figure 12), which is reinforced with sutures with heavy non-absorbable sutures, to create a circle (Figure 13). The graft is then inlayed through the extensor apparatus from both sides of the patellar tendon while converging in the quadriceps tendon (Figure 14), followed by suturing with heavy non-absorbable sutures (Figure 15). The augmentation is performed in full extension and can be adjusted to the underlying defects or procedures, thus allowing for additional sutures and reinforcements in each case.

The final construct is then abundantly irrigated and covered with the available soft tissues.

Our usual rehabilitation program starts with a brace in extension and no weight-bearing for 6 weeks. At 6 weeks, the patient starts physiotherapy and partial weight-bearing 25 kg per week with an allowed range of motion of 0–90°.

## Discussion

Augmentation for the treatment of extensor apparatus injury arose from the limited results of isolated direct repair and reconstruction with allografts or autografts [2].

Browne et al. performed a long-term study on allograft reconstruction for extensor mechanism failure following total knee arthroplasty, which showed a 38% failure rate and a 10-year survival rate of close to 50% [9].

The first report of the applicability of the Dacron graft belongs to Levin in 1976, which reported reconstruction of a chronic patellar tendon disruption with the aortic Dacron graft [10]. Since then, only scarce reports are available in the literature, with multiple materials, techniques, and applications: in 1980, Frasier repaired Achilles and patellar tendon tears in 9 patients with 5 mm Dacron vascular grafts underlining the possibility of earlier mobilization with this graft [11].

Levy et al. also used vascular Dacron grafts for quadriceps and patella tendons repairs without post-operative immobilization and reported good results in all patients [12].

More recently, different types of Dacron grafts (i.e., Dacron tape) have been described in the literature. Singh et al. report a variation with a double-eight fashion augmentation with a 6 mm Dacron graft in complex ruptures of the quadriceps, once again recognizing the earlier post-operative mobilization [13]. Fernandez-Baillo et al. described a primary suture repair augmented with Dacron tape, with excellent results at 1-year follow-up [14]. Bickels et al. also used Dacron tape to reconstruct the extensor mechanism after a proximal tibia endoprosthetic replacement in 55 patients, with positive results regarding restoration of extensor capacity [15]. Despite using the same material in our study, the surgical techniques and post-operative rehabilitation programs differ from ours.

Dacron vascular grafts, like the one used in our technique, are woven with PET (polyethylene terephthalate) yarn and impregnated with bovine origin collagen, which minimizes bleeding during implantation. The prosthesis is sterilized by irradiation and has a patented weaving process with textured yarns, excellent handling, remarkable elasticity, and structural strength. Because of their applicability in vascular surgery, these grafts have good healing properties, minimal fraying, and excellent seam retention. In the case of extensor mechanism augmentation, the bifurcated format is advantageous, permitting fine adjustment to the extensor apparatus from the tibial tuberosity to the quadriceps tendon. The main downside is the lack of biomechanical studies for its applicability in the musculoskeletal system.

Recently, three reports describe a successful technique using a synthetic Mesh [3, 16, 17] for extensor apparatus injuries with a sample of 77 patients from Mayo Clinic. Compared to Dacron techniques, the Mesh reports describe a longer immobilization period with a long leg cast (10–12 weeks) and less applicability since the cases described are all related to TKR injuries.

Ginesin et al. described a surgical technique for reconstructing chronic patellar tendon ruptures using an Achilles tendon allograft augmented with a vascularized hamstring tendon autograft and additional FiberTape augmentation, underlining the importance of augmentation in these injuries for additional stability, especially in the early rehabilitation [18].

Several augmentation techniques have been described to improve the integrity of extensor apparatus repairs, including using cerclage wires, Figure 8 sutures, tendon autografts or allografts, and multiple synthetic materials [2, 7, 19, 20], but standardized guidelines are lacking [6, 19, 20] despite proven superiority against isolated sutures in biomechanical and cadaveric studies [6, 7].

We provide a new construct design for this synthetic augmentation, with a wide range of possible applications, from acute tendon ruptures to complex revision cases or patella realignment procedures. The technique was initially developed to aid complicated cases, as shown in Table 1, but since then, the results have motivated us to expand indications, even in simpler procedures of the extensor apparatus searching for improved healing and faster rehabilitation. Primary injuries can provide augmentation to direct sutures or tendinous reinsertions, allowing for earlier recovery and extra confidence in the procedures. Secondary injuries (re-ruptures, fixation failures, periprosthetic injuries, or sequelae) with soft tissue retraction or deficiency benefit from the constitutional properties of the graft, functioning as an augment to repairs but also a substrate for soft tissue coverage and restoration while allowing for antibiotic impregnation in high-risk cases.

This straightforward technique has wide applicability and reproducibility, especially for surgeons familiar with knee surgery, with the possibility of usage in centers without access to allografts and patients for whom we are reluctant to harvest autografts.

With this in mind, we emphasize the wide range of possibilities that this procedure can offer in a variety of pathologies and complications, as well as we hope to contribute to a continuous investigation of the technique and product, aiming at increasing the reproducibility and reducing its costs.

Nevertheless, it is still unclear which surgical treatment is the gold standard since small sample sizes, heterogeneity, and inadequate follow-up in published case series are obstacles to a meaningful analysis [8].

## Conclusion

Dacron vascular graft applicability in extensor apparatus injuries has been scarcely reported in the literature since the 70s. Despite different indications and techniques, it can be combined with direct suture, Z plasty of the patellar or quadriceps tendon, allograft reconstruction, or MPFL reconstruction without interference or prejudice to the primary procedure. It provides a secure augmentation that allows earlier rehabilitation and mobility while adding strength and security to the overall construct, with several descriptions of good results with earlier mobilization and consistent range of motion. We present our augmentation technique for extensor apparatus injuries with promising results that should open the door to future research and optimization.

## Declarations

No benefits in any form have been received or will be received related directly or indirectly to the subject of this article.

On behalf of all authors, the corresponding author states that there is no conflict of interest to declare.
